# Estimation of the Closest In-Path Vehicle by Low-Channel LiDAR and Camera Sensor Fusion for Autonomous Vehicles

**DOI:** 10.3390/s21093124

**Published:** 2021-04-30

**Authors:** Hyunjin Bae, Gu Lee, Jaeseung Yang, Gwanjun Shin, Gyeungho Choi, Yongseob Lim

**Affiliations:** 1 Daegu Gyeongbuk Institute of Science & Technology (DGIST), College of Transdisciplinary Studies, Daegu 333, Korea; jinny3559@dgist.ac.kr (H.B.); leegu24@dgist.ac.kr (G.L.); yjs6813@dgist.ac.kr (J.Y.); shinkansan@dgist.ac.kr (G.S.); 2 Department of Interdisciplinary Engineering, Daegu Gyeongbuk Institute of Science & Technology (DGIST), Daegu 333, Korea; 3 Department of Robotics Engineering, Daegu Gyeongbuk Institute of Science & Technology (DGIST), Daegu 333, Korea

**Keywords:** alignment of point clouds to images, sensor fusion, bird’s eye-view (BEV), closest in-path vehicle (CIPV), autonomous emergency braking (AEB) test

## Abstract

In autonomous driving, using a variety of sensors to recognize preceding vehicles at middle and long distances is helpful for improving driving performance and developing various functions. However, if only LiDAR or cameras are used in the recognition stage, it is difficult to obtain the necessary data due to the limitations of each sensor. In this paper, we proposed a method of converting the vision-tracked data into bird’s eye-view (BEV) coordinates using an equation that projects LiDAR points onto an image and a method of fusion between LiDAR and vision-tracked data. Thus, the proposed method was effective through the results of detecting the closest in-path vehicle (CIPV) in various situations. In addition, even when experimenting with the EuroNCAP autonomous emergency braking (AEB) test protocol using the result of fusion, AEB performance was improved through improved cognitive performance than when using only LiDAR. In the experimental results, the performance of the proposed method was proven through actual vehicle tests in various scenarios. Consequently, it was convincing that the proposed sensor fusion method significantly improved the adaptive cruise control (ACC) function in autonomous maneuvering. We expect that this improvement in perception performance will contribute to improving the overall stability of ACC.

## 1. Introduction

In autonomous vehicles, collision assistance and avoidance systems for preceding vehicles are very important systems, and many researchers have been conducting much research related to these topics. The first step in preceding vehicle collision assistance or avoidance systems is the awareness of the vehicle ahead. For this perception process, various sensors such as LiDAR, radar, cameras, and GPS are used. In the case of using only the camera, object detection shows accurate classification performance, but there is a limitation in estimating the distance, speed, and coordinates of an object. On the contrary, in the case of using only LiDAR, it shows excellent results in performance such as the estimation of the distance, coordinates, and speed of an object, but the classification performance is significantly reduced. Moreover, if only radar is used, it shows superior performance in detecting objects at a longer distance than LiDAR, but there are still difficulties in classification. Each sensor has various ranges and limitations in recognizing the position, distance, and speed of an object.

Recent research related to autonomous vehicles has proposed various methods to increase the strengths of each sensor and complement each shortcoming through the sensor fusion process. In particular, in the fusion of camera data and LiDAR data, much research has been conducted to calibrate the point cloud of LiDAR with the camera image. There are various types of sensor fusion depending on where the data are projected. For studies using a bird’s eye-view (BEV), there is the fusion method of cameras and LiDAR by using the deep learning technique after calibration [1]. In many studies, fusion is performed by projecting the point cloud of LiDAR onto the image space. In this process, the intrinsic and extrinsic matrices of the camera are obtained by using a checker board, and the point cloud of LiDAR is projected onto the image by using this matrix [2,3]. Recently, instead of a general checker board, an image with a pattern has been used [4], and several checker boards have also been used [3,5]. A method of automatic calibration using three checker boards has been studied without placing checker boards of different sizes in various positions of one image [6].

In this paper, we verified the performance by applying the processed data to adaptive cruise control (ACC) using the newly proposed sensor fusion method. ACC requires the work of sending a signal to a lower level controller that determines the movement of the vehicle such as the brakes and engine from the upper level controller, which makes judgments from the data received from the sensor [7]. For ACC, PID control, model predictive control (MPC), and fuzzy logic control (FLC) algorithms are mainly used [8,9,10]. As a classical algorithm, PID control has features that are easy to implement, and MPC requires both accurately implemented models and having much necessary information. In the case of FLC, it has the characteristic that it uses a larger number of parameters than PID control [11].

For the high performance of ACC, it requires accurate distance information of the preceding vehicle. For an index for evaluating the performance of ACC, the International Organization for Standardization (ISO) presented the performance of recognizing the distance to the preceding vehicle on a straight road, the accuracy of recognizing the vehicle ahead, and the performance of recognizing the vehicle ahead on a curved road [12]. In particular, various situations occur depending on the path where the ego vehicle intends to go through or when overtaking the preceding vehicle. Thus, various methods for accurately recognizing the situation in such a complex situation are being studied. Among them, there is also a study to detect the closest in-path vehicle (CIPV) in various scenarios using a multi-class support vector machine (SVM) and a radar system [13]. In other words, the ACC algorithm is also a promising research topic, but it is important to receive information about the nearest vehicle in front of the vehicle path before determining the control inputs. Therefore, the main process is to combine the information about paths or detected objects that can be obtained from multiple sensors. Although in detecting an object by using LiDAR, there is a limitation in the distance because the light transmittance varies depending on the color or material of the vehicle. The 3D data and wide field-of-view (FoV) of LiDAR are useful for recognizing the surrounding environment in a city, and this will have many uses in future autonomous driving research works.

In this paper, we also proposed a new method to increase the accuracy of the detection of the CIPV at a middle distance by the method of the sensor fusion of the object tracking results of the low-channel LiDAR and the object tracking results of the vision sensor. We experimentally show the improved results in the case of applying the proposed algorithm to the AEB test. In order to perform a fusion of LiDAR and camera tracking data, a checker board was used to obtain the extrinsic and intrinsic parameters of the camera, and then, we used these to project the data; however, this is a cumbersome task that depends on the size, location, and sensor location of the checker board. In particular, it is not easy to use a checker board when LiDAR has a small number of channels. Therefore, we also proposed a method of the fusion of the two data through the IoU after experimentally finding the value used in the equation to properly project the 3D LiDAR data on a 2D image without using a checker board. Afterwards, the data detected through the camera were converted into a BEV by inversely using the equation used to project the LiDAR onto the image. Subsequently, ego vehicle path information was also used to find the CIPV by projecting it onto the BEV space. This obtained CIPV information is applied to ACC. Finally, in order to ensure that this set of processes is well aware of multiple road conditions, we demonstrated the experimental results in various scenarios, such as straight roads, side lanes, curves, and intersections.

In this study, we proposed a new sensor fusion method utilizing the object tracking results obtained from LiDAR and camera sensors. It also informs whether the recognized object is the CIPV of the ego vehicle so that the fusion result can be applied to ACC. As a result of applying this to ACC, we finally were able to improve its performance. The main contributions of this study are as follows:

- We proposed an empirical projection method in which the LiDAR points to the 2D image and used the IoU to fuse the two sensors’ data. The proposed method showed good performance with a low-channel LiDAR.
- We proposed a BEV estimation and CIPV calculation method of the objects (e.g., cars) using vision and low-channel LiDAR data.
- We validated our method in a real-world situation through the AEB test. We showed that the CIPV through the proposed method contributed to improving the performance of AEB.

This paper is organized as follows: Section 2.1 shows the sensor setup and specifications. Section 2.2 demonstrates the object tracking of the LiDAR and the distance accuracy of the tracked data. Section 2.3 presents the distance estimation with the detected object bounding box height and simple tracking algorithm. Section 2.4 shows the equation to align the image and LiDAR points and transform the pixel image coordinates to the BEV coordinates. Section 2.5 and Section 2.6 show the algorithm for each fusion and ACC. Section 3 presents the performance of the estimation the CIPV and ACC in various scenarios and provides discussions.

## 2. Materials and Methods

### 2.1. Test Environment

#### 2.1.1. Sensors’ Description

**Figure 1:**
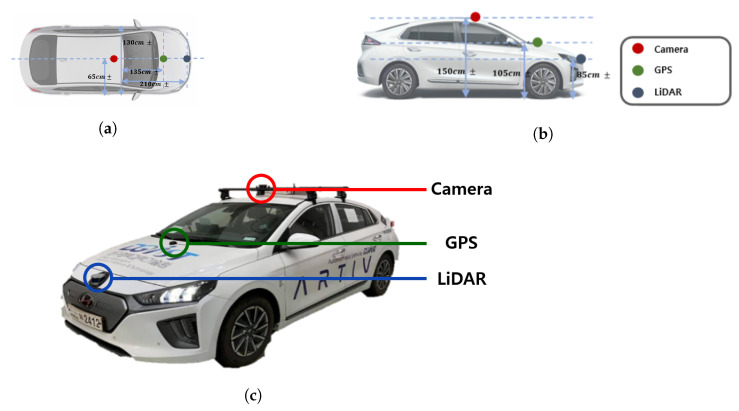
Sensors’ configuration: (**a**) Vehicle top view and sensor location. (**b**) Vehicle side view and sensor position. (**c**) Sensor installation on the test vehicle.

Table 1 shows the sensors’ specification that we used. The Puck LiDAR sensor with the lowest number of channels among the 360 degree LiDAR of Velodyne was used. The horizontal FoV of the Puck LiDAR is 360 degrees, and the vertical FoV is 30 degrees (i.e., −15 degrees to +15 degrees). The resolution is 2 degrees. Although it is stated that it can measure up to 100 m in the specifications, when viewing actual point cloud data using the visualization tool provided by Velodyne, points over 60 m have a limit in that it is difficult to identify objects. In addition, since the LiDAR is embedded in the front bumper of the vehicle, the FoV utilized in this test was 180 degrees instead of 360 degrees. The camera sensor used in this experiment was the Logitech StreamCam. The resolution is 720p and 60fps, and it has an FoV of 78 degrees vertically. The vehicle used in the experiment was a modified vehicle to which was applied the drive-by-wire system, the HYUNDAI Ioniq electric vehicle, and each sensor was installed as shown in Figure 1.

#### 2.1.2. Proving Ground for the Experiments

**Figure 2:**
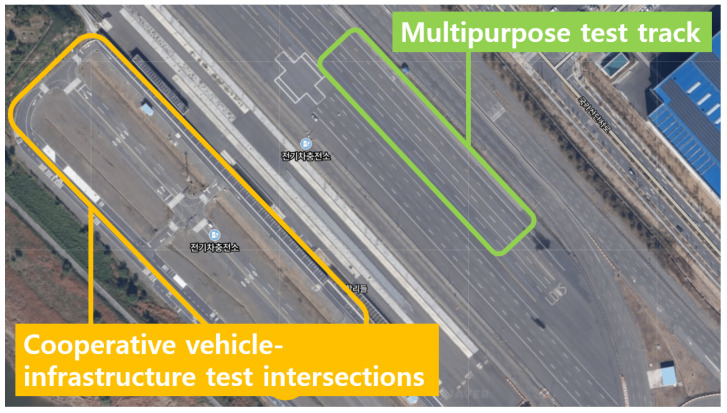
Satellite photo of the test environment at KIAPI.

The vehicle test was conducted at the Korea Intelligent Automotive Parts Promotion Institute (KIAPI), located in Daegu, Korea. Tests for various scenarios were performed using a multipurpose test track capable of braking-related tests and a cooperative vehicle-infrastructure test, as well as an intersection test through various road environments. Figure 2 shows the actual appearance of the facility.

### 2.2. LiDAR Object Tracking

#### 2.2.1. Point Cloud Segmentation and Tracking

The main objectives by collecting and analyzing point cloud information of objects obtained by the LiDAR were to find their size, location, and velocity. Segmentation and tracking of objects were implemented through an open source [14]. Since points belonging to the same channel were given as more data than necessary in the point cloud, the downsampling process was performed to reduce the number of points by a certain percentage.

Point cloud segmentation was divided into ground selection and non-ground object detection, respectively. The ground plane fitting was used to select the ground. This algorithm performs faster than RANSAC because there is no random sample selection process [15]. The algorithm was applied by dividing the area by the point cloud according to the vehicle movement direction.

This algorithm needs constants $N_{iter}$, $N_{lowestpointrepresentative}\left(N_{LPR}\right)$, $Th_{seeds}$, and $Th_{dist}$ as inputs. $N_{iter}$ is a constant that sets the number of times to execute the fitting. The average of several points was used to obtain a plane with reduced errors due to noise, where $N_{LPR}$ is a constant regarding how many reference points were set. $Th_{seeds}$ is a constant that sets the initial seed threshold of the ground using LPR. $Th_{dist}$ is a constant for the next iteration by changing the plane expression by adopting a point that is smaller than $Th_{dist}$ from the calculated plane as the ground. Setting the approximate height of the ground seed by using $Th_{seeds}$ arbitrarily set in the initial point cloud was done. After this procedure, initialized $N_{LPR}$ points at the settled height as the seed were obtained. In the first iteration, the plane was inferred as the seed. The simplified calculation process was achieved by setting the plane with linear Equation (1) as follows.

(1) $$n_{1}x+n_{2}y+n_{3}z+n_{4}=0$$

where *n* is the coefficient representing the plane and *x, y, z* are the coordinates of the point. The covariance matrix *C* of the 3D point selected as the seed was calculated with the aim of obtaining a linear plane that was fitted most appropriately to the ground truth.

(2) $$C=\sum_{P_{seed}}\left(p_{i}-\hat{p}\right){\left(p_{i}-\hat{p}\right)}^{T}$$

where the covariance was obtained by squaring the difference between each seed point (i.e., $p_{i}$) and the mean of the seed point (i.e., $\hat{p}$), then taking the summation of all of these. By applying SVD to the covariance matrix obtained from Equation (2), the plane with the least covariance was adopted as the linear plane model. A point with a distance smaller than $Th_{dist}$ from the plane model was added to the seed and used as a seed point to infer the plane in the next iteration, and the other point was distinguished as non-ground. Finally, we performed this process $N_{iter}$ times to proceed with the ground segmentation.

Euclidean cluster extraction [16] provided by the point cloud library (PCL) was applied to detect objects from non-ground points that were identified above. The point cloud on 3D coordinates was represented by a KD-tree based on the location of points to find the nearby points [17]. When clustering all points, a queue was created. In other words, these points with a distance smaller than the threshold were added to the queue, and points that were already included in other clusters were ignored. However, when there were no more nearby points, the queue just started to form a cluster. Due to the characteristics of the LiDAR, the distance between each channel of the LiDAR increased as the distance increased. In the 16-channel LiDAR used in this study, there was a tendency that points with a long distance in the z-axis direction were not recognized as the same object. Accordingly, the performance was improved by assigning a weight to the distance about the z-axis between points.

Point cloud tracking was performed by receiving the object point size and position obtained from the segmentation. The segmented object was tracked by calculating the Gaussian uncertainty estimated with the Bayesian filter [18].

(3) $$p\left(x_{k}|x_{k-1},x_{k-2},\ldots ,x_{1},x_{0}\right)=p\left(x_{k}|x_{k-1}\right)$$

In recursive Bayesian estimation, Equation (3) was applied to the object tracking using the idea that the immediately previous state (i.e., $x_{k-1}$) was conditionally independent of the other previous states (i.e., $x_{k-2},\ldots ,x_{1},x_{0}$) from the probability of the given current state (i.e., $x_{k}$) due to the Markov assumption. Since the state of the object from the previous frame was saved, the nearest object that had a similar number, density, and distribution of points and that was less than the set threshold for the Euclidean distance was determined as the same object in the next frame. The previous and current states of this object were connected by a trajectory. During the above process, a new track was added while a track was maintained, and a track was deleted, respectively. According to both the passage of time and the moving direction of the segmented new object, the condition of tracking the objects was completed only when the space where the object existed was empty. However, if an object did not have a trajectory, the process of adding a track was required. Furthermore, if the trajectory could not be connected by comparing the previous certain frames, the track was deleted. Moreover, if the trajectory could be connected to the object, the track was maintained. Subsequently, since the trajectories obtained through the above processes were able to be expressed as vector values, the velocity of the objects could be obtained.

Information given from the point cloud of the tracked object was assigned as the ID of the object, the x, y, and z length of the object, the x, y, and z distance from the LiDAR to the nearest object point, and the relative speed for each x-, y-, and z-axis, respectively.

#### 2.2.2. Distance Accuracy from Tracked Data

In order to check the error rate of the data obtained from the algorithm, a test was conducted to compare the ground truth and the tracked data. The ground truth was set by attaching the GPS to the position closest to the LiDAR on the object, and the result was obtained close to the point closest to the object.

**Figure 3:**
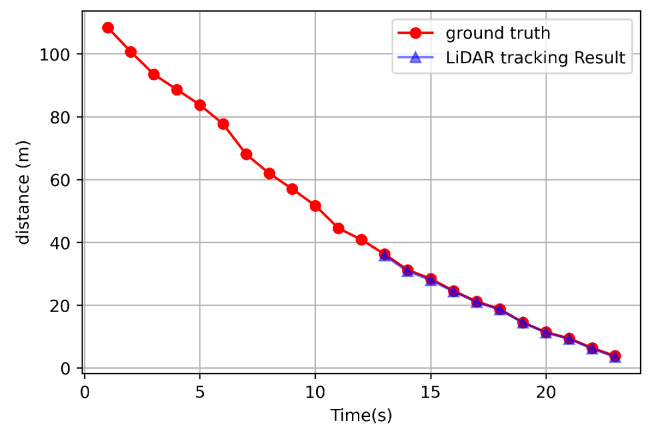
Distance comparison between the ground truth and from tracked data.

Figure 3 shows that the distance comparison of an object’s distance could be obtained through point cloud object tracking and the ground truth. However, object tracking was not possible at a distance greater than about 35 m, so only the results of 0–35 m are shown in Figure 3. In other words, Figure 3 compares the result while the target vehicle moved in the direction of the ego vehicle little by little from a distance of 35 m. Moreover, as shown in Figure 3, as a result of calculating the actual error rate, the rate of 2.14% was obtained. In particular, an error distance of 0.47 m occurred at the largest distance. This algorithm was executed for 15–20 milliseconds based on the ros time when there were 10 objects around the LiDAR.

As a result of the experiment, the LiDAR was able to obtain accurate results within 35 m, but it was confirmed that the vehicle was not able to be recognized beyond 35 m. Therefore, this was the main reason why the sensor fusion was required.

### 2.3. Vision Object Tracking

#### 2.3.1. Object Detection

The deep learning network model was also used for object detection in this study. Among various object detection models, YOLOv3, which shows high speed and high accuracy, was used [19]. In order to increase the real-time performance of object detection, NVIDIA’s TensorRT was also applied. The result obtained through this process included information of the class, confidence, and x1,y1,x2, and y2 per object.

#### 2.3.2. Distance Estimation with Regression

**Figure 4:**
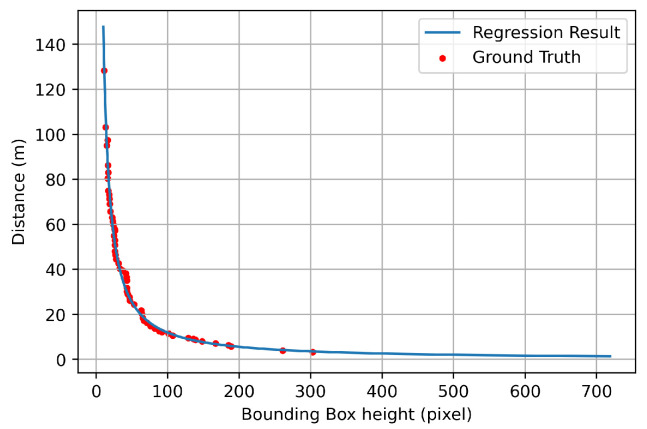
Distance comparison results between the ground truth and estimation through regression.

**Figure 5:**
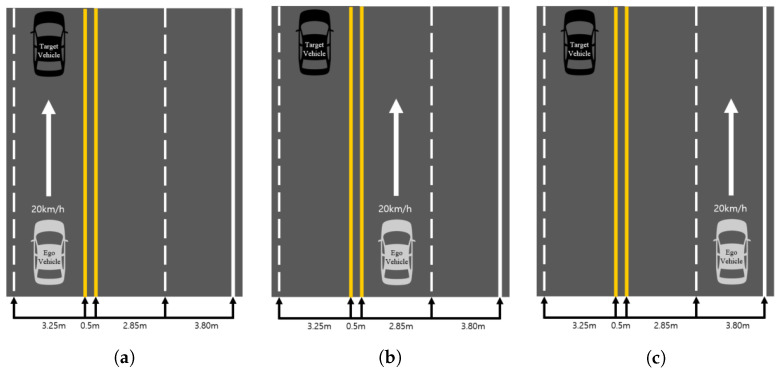
Distance estimation test scenarios. Scenarios (**a**–**c**) represent the longitudinal difference that can occur in real road environments.

For the BEV transform process, the distance was estimated when the detected object was recognized as a vehicle. For this case, power regression was performed by using the height information of the bounding box and the actual distance value obtained from the GPS. At this time, only the height information of the bounding box was used to obtain a constant result regardless of the current direction (i.e., front and side) of the vehicle. The result is shown in Equation (4) and Figure 4.

(4) $$d_{est}\left(h_{bbox}\right)=1829.1\cdot h_{bbox}^{-1.093}$$

where $d_{est}$ is the estimated distance and $h_{bbox}$ is the height of the detected object bounding box. By using this, the distance could be estimated using only the height information of the bounding box. For the regression results are shown in Figure 4, the red dot is the data measured at various distances, and the blue line is the result of the regression. Moreover, in order to check the distance estimation results using Equation (4) in various situations, three scenarios as shown in Figure 5 were tested.

**Figure 6:**
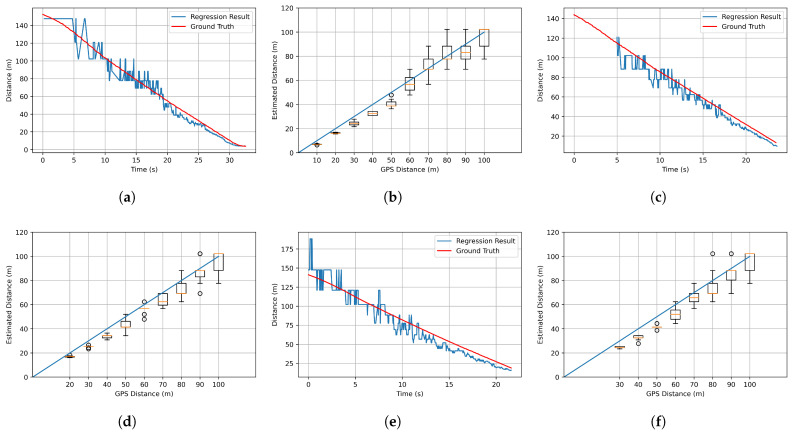
Distance estimation test result. (**a**,**c**,**e**) are the results of testing in the situations of Figure 5a–c. The ground truth (from GPS) and estimated distance are plotted together in the situation of the ego vehicle moving toward the stopped target vehicle. The x-axis is time, and the y-axis is the estimated distance between the ego vehicle and the target vehicle. (**b**,**d**,**f**) are the results of repeating the situations of (**a**,**c**,**e**) 20 times. The x-axis is the ground truth (from GPS), and the y-axis is the estimated distance between the ego vehicle and the target vehicle.

As shown in Figure 5, a scenario that can represent a possible horizontal error in road conditions is constructed. The scenario in Figure 5a is that target vehicle and the ego vehicle are in the same lane. The scenarios in Figure 5b,c are in the next lane or farther away.We wanted to see if there was any difference in the performance measured when the vehicle was further away in the horizontal direction. The test results in each situation shown in Figure 5 are demonstrated in Figure 6. Figure 6a,c,e shows the results of plotting the estimated distance using Equation (4) and the actual distance measured using the GPS in all the situations shown in Figure 5. The statistical results showing the median, minimum, and maximum value of each of the 20 attempts are also shown in Figure 6b,d,f.

At this time, as the distance between the ego vehicle and the target vehicle increased, the consistency of the size of the recognized bounding box decreased. Thus, this result had a significant impact on the results of distance estimation. This is a chronic problem with YOLOv3, the one-stage object detector that we used, showing a result in which this algorithm was not able to recognize small objects well. In addition, since the detection result of YOLOv3 is not continuous, the estimated distance severely fluctuated. To solve this problem, the binning technique was applied. In the case of more than a 60 m distance in Figure 6b,d,f, where the variance of each sample increased, outliers appeared frequently. For this reason, if the estimated distance was more than 60 m, the information was not used in the estimating process. The actual distance information was used by binning data from 5 m to 60 m with a 10 m incremental process.

Binning also caused an error between the estimated distance and the actual distance. However, when the distance between the ego vehicle and the target vehicle stayed at a long distance, the small error occurring in the distance estimation process was not significant. In the fusion process, if the distance between the ego vehicle and the target vehicle was sufficiently close, the distance information recognized by the LiDAR was used, not the distance estimated through vision. Therefore, we note that the above proposed approach was appropriate.

#### 2.3.3. Object Tracking

TensorRT was applied to YOLOv3 to increase the real-time object recognition, while this process caused a decrease in the accuracy of the model. Thus, we estimated the distance between the ego vehicle and the objects by using the information in the bounding box of the detected object as described in Section 2.3.2. Therefore, the flickering of the object detection result created a situation in which an obstacle appeared or disappeared suddenly in the autonomous vehicle, which led to rapid acceleration and deceleration. For this reason, we proposed a tracking algorithm that was able to continuously recognize objects using past information.

In this study, YOLOv3 was used for detection, and the situation in which classes other than cars were recognized was not considered. Inconsistent recognition performance was compensated through the proposed simple tracking algorithm (see Algorithm 1). This algorithm has a time complexity of O(nm), where n is the number of detected objects in the current frame and m is the size of T. The main idea is that objects are compensated using past information, even if they are not actually detected. GET_CANDIDATE_OBJECT() searches all *P* in *Q* and gets the currently used ID. After that, a list of candidate objects using the object information of the most recent frame corresponding to each ID is returned. CALCULATE_DISTANCE() calculates the actual distance between the ego vehicle and the detected object using Equation (4). This algorithm was executed within 4.5 milliseconds on average in the scenario in Figure 5. This execution time included the ros delay.
**Algorithm 1** Simple tracking algorithm.
     **Input** list of objects (*O*)
     **Output** list of objects with distance information (*O*)
1:$O\leftarrow \left\{o_{1},o_{2},\;\ldots \right\}\;(o_{i}$ is the object with class,confidence,x1,y1,x2,y2)2:$Q\leftarrow \left\{P_{1},\;\ldots ,P_{10}\right\}\;(Q$ is the queue; $P_{i}$ is the list of objects from the past frames)3:$dist_{th}\leftarrow$ (determined heuristically)4:**if** all *P* in *Q* are none **then**                   ▹ if *O* is the first frame5:    **for all** $o_{i}$ in *O* **do**6:        $o_{i}\cdot ID\leftarrow \mathrm{newID}$7:        $o_{i}\cdot distance\leftarrow CALCULATE\_DISTANCE\left(\left(o_{i}\cdot y_{2}-o_{i}\cdot y_{1}\right)\right)$8:    **end for**9:**else**                           ▹ if *O* is not the first frame10:    T←GET_CANDIDATE_OBJECT()11:    **for all** $o_{i}$ in *O* **do**12:        $o_{i}\cdot distance\leftarrow CALCULATE\_DISTANCE\left(\left(o_{i}\cdot y_{2}-o_{i}\cdot y_{1}\right)\right)$13:        **for all** $t_{j}$ in T **do**14:           L2 distance $\leftarrow \sqrt{{\left(\frac{o_{i}\cdot x_{2}-o_{i}\cdot x_{1}}{2}-\frac{t_{j}\cdot x_{2}-t_{j}\cdot x_{1}}{2}\right)}^{2}+{\left(\frac{o_{i}\cdot y_{2}-o_{i}\cdot y_{1}}{2}-\frac{t_{j}\cdot y_{2}-t_{j}\cdot y_{1}}{2}\right)}^{2}}$15:           **if** L2 distance < $dist_{th}$ **then**16:               $o_{i}\cdot ID\leftarrow t_{j}\cdot ID$17:               **break**18:           **end if**19:           $o_{i}\cdot ID\leftarrow new\;ID$20:        **end for**21:        **for all** $t_{j}$ in *T* **do**22:           **if** $t_{j}$ is not in *O* **then**23:               $O\cdot append(t_{j})$24:           **end if**25:        **end for**26:    **end for**27:**end if**28:Q·append(O)29:**return** *O*


### 2.4. Object 3D Coordinate Estimation

#### 2.4.1. The Alignment Method of the Camera and LiDAR Point Cloud

**Figure 7:**
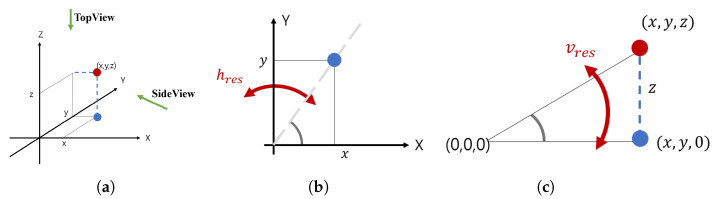
(**a**) Coordinate system of the LiDAR. (**b**) Top view and (**c**) side view of (**a**).

**Figure 8:**
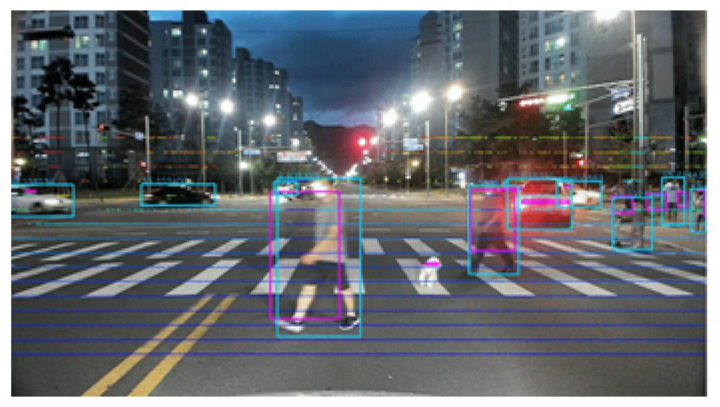
Alignment of the LiDAR bounding box and the YOLOv3 bounding box from the tracking result. The magenta color is the LiDAR bounding box, and the cyan color is the YOLOv3 bounding box.

(5) $$x_{img}=\frac{\arctan\frac{-y}{x}}{h_{res}}$$

(6) $$y_{img}=\frac{\arctan\frac{z}{\sqrt{x^{2}+y^{2}}}}{v_{res}}$$

where $h_{res}$ and $v_{res}$ mean the horizontal resolution and vertical resolution. As shown in Figure 7, both the LiDAR point cloud and the equation were used to project the cuboid of the bounding box, which can be obtained through the LiDAR object tracking result, onto the image. $x_{img},y_{img}$ were obtained through projection by using Equations (5) and (6). With larger $h_{res}$, a larger horizontal gap between the dots on the image was obtained. As $h_{res}$ became smaller, the horizontal gap between the points decreased. For $v_{res}$, it also worked the same for vertical spacing. In the paper, $h_{res}$ and $v_{res}$ in the above equation were experimentally adjusted so that the image and LiDAR point were well aligned. The result of actually projecting the LiDAR point clouds and bounding boxes onto the image using the above equation is shown in Figure 8.

As shown in Figure 8, the result of the projection of the bounding box and point cloud of the LiDAR matched the image well by using Equations (5) and (6). In particular, Figure 8 shows high accuracy despite the situation where people and cars are moving fast.

#### 2.4.2. Transforming the Image Pixel to the BEV

The equation used to project LiDAR point clouds onto the image was used inversely to transform $x_{img}$ and $y_{img}$ into x and y.

(7) $$\begin{array}{cc} x & =\sqrt{x^{2}+y^{2}}\cos\left(x_{img}h_{res}\right)\\ \; & \approx d^{*}\cos\left(x_{img}h_{res}\right) \end{array}$$

(8) $$y=x_{img}\tan\left(x_{img}h_{res}\right)$$

where $x_{img}$ and $y_{img}$ are the pixel image coordinates, while *x* and *y* are the BEV coordinates. We used Equations (7) and (8) to convert the bounding box through the tracking result of vision into the BEV coordinates. In particular, the values of $h_{res}$ and $v_{res}$ were the same as the values used in Figure 8. In this paper, the purpose of this transform was to calculate the BEV when vision recognized the vehicle even in the range that the LiDAR was not able to detect objects. When only vision recognized the target vehicle, both $x_{img}$ and $y_{img}$ were known. Therefore, the object distance using regression was estimated. Finally, the distance $d^{*}$ obtained through estimation was substituted for $\sqrt{x^{2}+y^{2}}$, and the x,y value of the BEV was calculated by using Equations (7) and (8).

**Figure 9:**
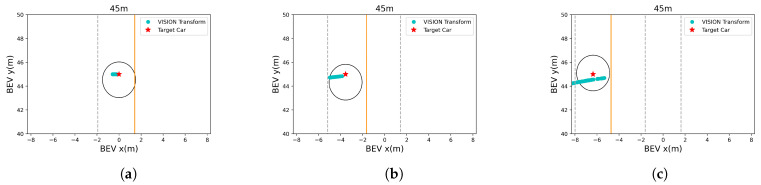
BEV transform result from the distance estimation. The ego vehicle is located at (0,0). The yellow and gray dotted lines in the graph represent the yellow and white lanes shown in Figure 5. (**a**) Result of the scenario in Figure 5a. (**b**) Result of the scenario in Figure 5b. (**c**) Result of the scenario in Figure 5c.

**Figure A1:**
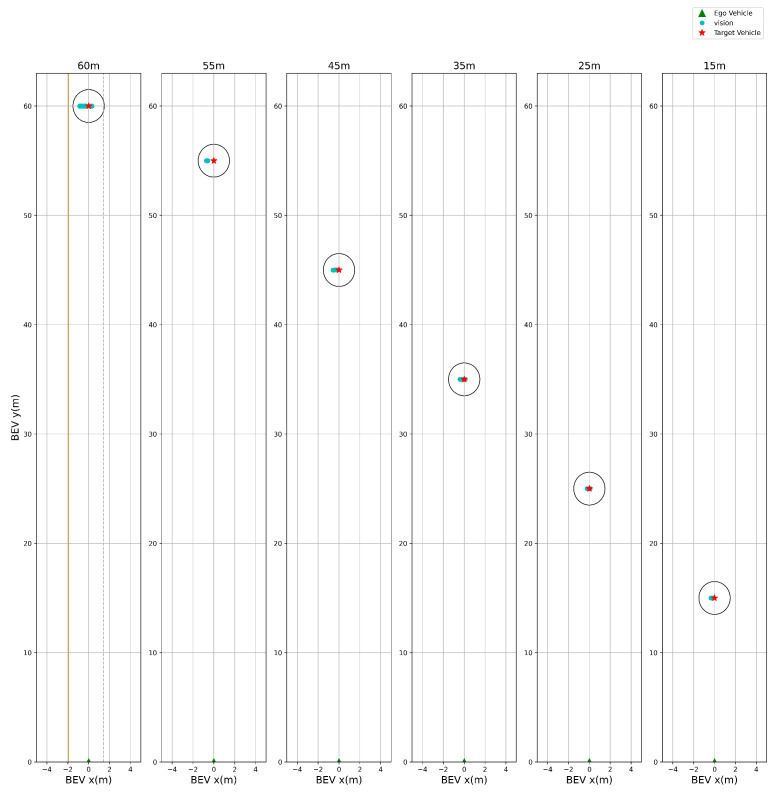
The BEV transform result of the scenario in Figure 5a. This is the result of transforming the pixel image coordinates of the YOLOv3 bounding box by distance into the BEV. The radius of the circle in the figure is 1.5 m, which is half of one lane width.

**Figure A2:**
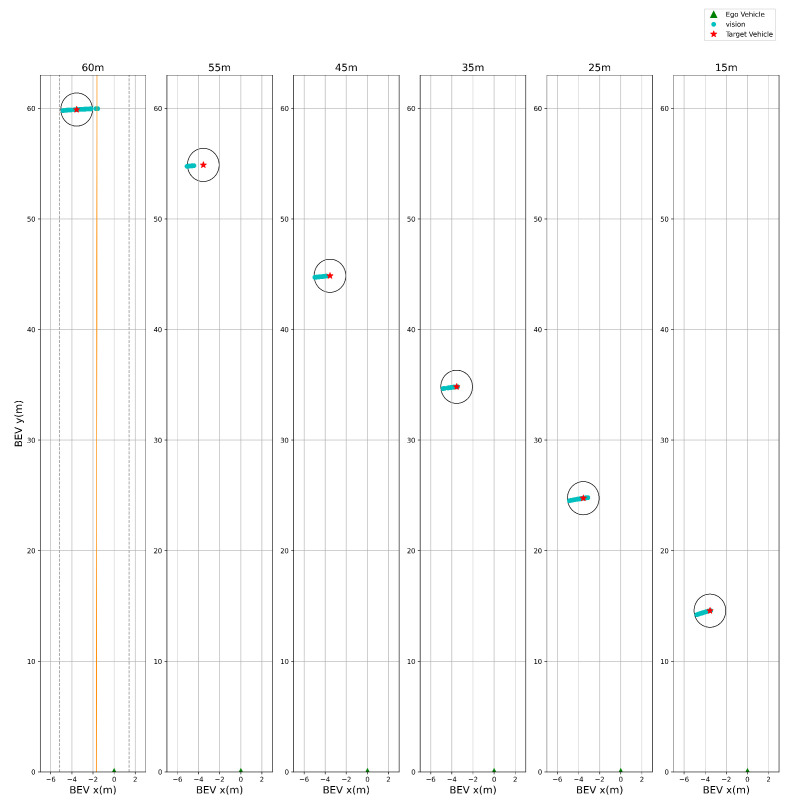
The BEV transform result of the scenario in Figure 5b. This is the result of transforming the pixel image coordinates of the YOLOv3 bounding box by distance into the BEV. The radius of the circle in the figure is 1.5 m, which is half of one lane width.

**Figure A3:**
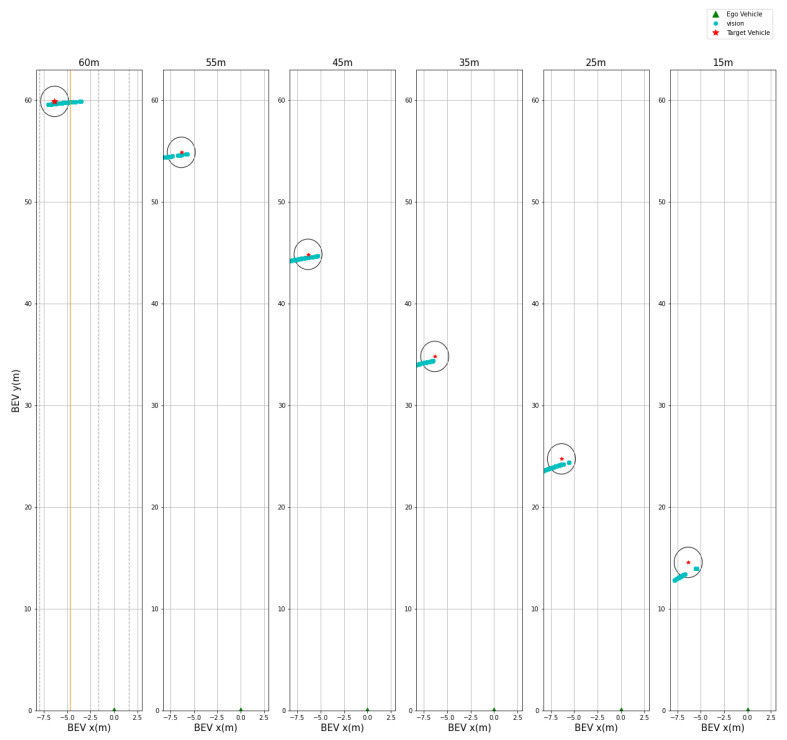
The BEV transform result of the scenario in Figure 5c. This is the result of transforming the pixel image coordinates of the YOLOv3 bounding box by distance into the BEV. The radius of the circle in the figure is 1.5 m, which is half of one lane width.

We validated whether the obtained BEV result was correct by using the three scenarios shown in Figure 5. As shown in Figure 5, the tracking results of YOLOv3 were achieved by moving the ego vehicle closer and closer to the target vehicle from a distance of 100 m or more. Then, among them, *x* and *y* were calculated by substituting the image pixel coordinates of the bounding box recognized at a distance of 45 m from the trace into Equations (7) and (8). x and y are the cyan dots of Figure 9 on the graph. As shown in Figure 9, the lateral error was 1.5895 m, and the longitudinal error was 0.7408 m. These results show that both the lateral and longitudinal errors were at the level of discriminating lanes and thus were sufficiently accurate to use the CIPV. Figure 9 shows the results when the actual distance was 45 m. However, due to space limitations, other figures at different target distances are shown in the Appendix: Figure A1, Figure A2 and Figure A3. There was a tendency that the lateral error increased from Figure 9a–c. From Figure 9a–c, the location of target vehicle was located at the far left of the image. The distortion of the image was more severe, and the alignment was not well aligned with that of the center part of the image, so the error of the BEV transform was slightly greater. However, since the error value was actually moving within the width of one lane, it was also considered as a sufficiently low error level to distinguish the target vehicles in the path of the ego vehicle.

### 2.5. Fusion of LiDAR and VISION

**Figure 10:**
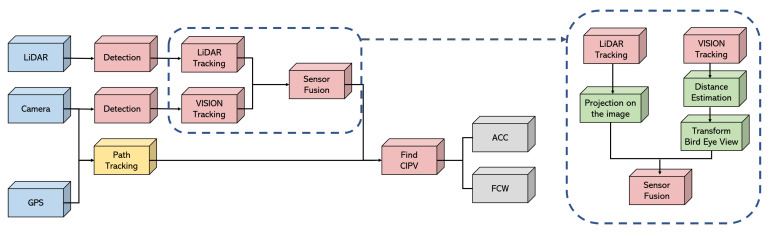
Process flow of fusion for ACC application.

In this paper, the various experiments were conducted using the LiDAR, camera, and GPS, and Figure 10 shows how the LiDAR and camera data were fused and the processing procedures after fusion. In particular, we fused both the object tracking results from the LiDAR and the object tracking results from the vision camera. There was no distance and BEV coordinate data in the object tracking result by vision. Therefore, as shown in the box with the dashed line in Figure 10, the distance was estimated before fusion, and the BEV transform was performed by using the result. After that, the cuboid bounding box obtained through the object tracking result of the LiDAR was projected onto the image, and the fusion was performed by comparing it with the bounding box obtained through the object tracking result of vision.

#### 2.5.1. Fusion of the Camera and LiDAR Tracking Data with the IoU

The IoU was calculated and compared between the bounding box tracked through vision and the bounding box tracked through LiDAR. Let *n* be the number of objects tracked by vision and *m* be the number of objects tracked by LiDAR. Then, the IoU value when each box is overlapped is put into an n×m matrix. After that, values are added to each column and row, and if the added value in each column is zero, only vision is recognized, and the case isa non-zero case. If the value of row *j* in column *i* is the same as the maximum value among the values in column *i* while moving rows one by one, it is considered as a case where LiDAR and vision recognize the same object together. The remaining cases were considered as cases where only LiDAR was used to detect objects. Since object data tracked by LiDAR and object data tracked through vision have the coordinates of the bounding box, the IoU was calculated using the coordinates, and then, the form of the fusion data (see Table 2) was obtained through Algorithm 2.
**Algorithm 2** Fusion algorithm.
     **Input** object data tracked by LiDAR *L*, object data tracked by vision *V*
     **Output** *FusionData*
1:m←length(L)2:n←length(V)3:**for all **$L_{i}$ in L **do**4:    **for all** $V_{i}$ in V **do**5:        $\mathrm{IOU}\;\mathrm{matrix}\left[i,j\right]\leftarrow IOU\left(L_{i},V_{j}\right)$6:    **end for**7:**end for**8:**for all **$V_{i}$ in V **do**9:    **if** Sum(IOU matrix[i,0:m])iszero **then**10:        $\mathrm{FusionData}\leftarrow V_{i}$                       ▹ Only vision case11:    **end if**12:    **for all** $L_{j}$ in L **do**13:        **if** IOU matrix[i][m]ismax(IOU matrix[i][0:m]) **then**14:           $\mathrm{FusionData}\leftarrow V_{i}\&L_{j}$                  ▹ LiDAR + vision case15:        **else**16:           $\mathrm{FusionData}\leftarrow L_{j}$                       ▹ Only LiDAR case17:        **end if**18:    **end for**19:**end for**20:**return** FusionData


In Algorithm 2, IOU(A,B) calculates the IoU of A and B where A and B are rectangular bounding boxes. This fusion algorithm and the preprocessing such as the BEV transform were executed within three milliseconds. Though the algorithm depends on LiDAR tracking and YOLOv3 tracking, the execution time of the fusion algorithm was fast enough to be used in a real-time system.

#### 2.5.2. Result of Fusion Data

By projecting the LiDAR points onto the image, the bounding boxes of LiDAR and vision could be aligned on the same image coordinate system. After that, the bounding boxes of the two sensors were calculated by comparing the IoU between the boxes, and the case where each bounding box detected the same object was searched, then the tracked data were combined.

After the fusion of each bounding box, there were three cases: only vision, only LiDAR, and both vision and LiDAR. For a total of three cases, the 10 items shown in Table 2 are summarized. In the case of in-path information, a value of the Boolean type represents whether the BEV of the object is between the left and right lanes. Then, the BEV was used to obtain the CIPV.

### 2.6. ACC

ACC, a typical longitudinal control system applied to autonomous driving, was used to verify the performance of the newly proposed sensor fusion algorithm in this study. Based on the preceding car-following system in ACC, longitudinal control was performed by using the calculated risk by predicting the behavior of surrounding obstacles. Thus, this study demonstrated that the proposed sensor fusion algorithm based on better estimation of surrounding obstacles was able to improve the performance of ACC in diverse scenarios.

#### 2.6.1. Implementation of ACC

PID control was used to control the vehicle speed for the upper level controller for ACC [8]. It received the desired velocity as an input and started the control loop, then used the current vehicle speed as feedback. In the process of multiplying integral gain, an anti-windup process was added to prevent rapid acceleration due to previously accumulated errors. However, the scaling factors of the accelerator pedal value or braking pedal value are transmitted to the lower level controller. The jerk of the vehicle was not considered in the lower level control loop, as the timing of the initial CIPV recognition was important.

In order to validate distant object recognition and the fast response performance of sensor fusion, the vehicle speed was calculated by using a simple formula. The target vehicle speed of ACC using the data obtained from sensor fusion was calculated by Equation (9), which was simplified to a linear equation with reference to [20,21].

(9) $$v_{desired}=v_{current}\cdot \left(\frac{d_{current}-d_{min}}{d_{desired}-d_{min}}\right)$$

where $v_{desired}$ is the target speed, $v_{current}$ is the current speed of the vehicle, $d_{current}$ is the distance from the vehicle to the closest object on the driving path, $d_{desired}$ is the safety distance calculated based on the vehicle speed, and $d_{min}$ is the minimum safety distance.

As shown in Algorithm 3, ACC examines two conditions to understand the surrounding traffic conditions. In other words, it examines whether it is an object of the CIPV or an object approaching the vehicle via time-to-collision (TTC). In ACC, the CIPV data were used to follow the preceding vehicle, while the TTC data were used to respond to unexpected obstacles.

#### 2.6.2. AEB Test

The sensor fusion algorithm significantly contributes to improving the performance of the response to preceding vehicles in the case of a high risk of collision. Therefore, among the ACC test protocols, we verified whether the proposed sensor fusion algorithm was sufficiently effective by using the AEB test, which requires rapid deceleration. To verify the performance of sensor fusion, an AEB test scenario was used in the autonomous driving test conducted by Euro NCAP in 2020 [22].

1. Response to a vehicle in front in a stationary state: car-to-car rear stationary (CCRs)
2. Response to a vehicle in front at a slow speed: car-to-car rear moving (CCRm)
3. Response to a vehicle in front decelerating: car-to-car rear braking (CCRb)

**Algorithm 3** ACC algorithm.
     **Input** Fusion data *O* and vehicle current velocity $v_{current}$
     **Output** Vehicle desired velocity $v_{current}$
1:**for all** *O* **do**2:    **if** object is CIPV **then**3:        **if** $d_{current}\leq d_{min}$ **then**4:           $v_{desired}\leftarrow v_{current}\cdot \left(\frac{d_{current}-d_{min}}{d_{desired}-d_{min}}\right)$5:        **else**6:           $v_{desired}\leftarrow v_{current}$7:        **end if**8:    **else**9:        **if** Objects with TTC below the threshold **then**10:           Slow down until TTC goes below the threshold11:        **else**12:           $v_{desired}\leftarrow v_{current}$13:        **end if**14:    **end if**15:**end for**16:**return** $v_{desired}$


## 3. Experimental Results

### 3.1. Qualitative Evaluation

Table 3 demonstrates the result of the fusion data with Figure 5. Table 3 also presents the error between the BEV with fusion data and the real UTM coordinates of the target vehicle.

The error value was calculated by parsing the data recognized as a car among the data from the result of the fusion. The mean absolute error (MAE) is the average value of the absolute error of the BEV coordinates and GPS UTM coordinates. As shown in Table 2, the case of vision + LiDAR was the result of the fusion between the BEV data, which was the result of the LiDAR data, and the classification resulted as the vehicle, which was from the vision data. In the case of only vision, the data were the result of the transform to the BEV. In the result of Figure 5a, especially the vision + LiDAR type, the MAE in the distance error, lateral error, and longitudinal error values were smaller than the horizontal and vertical size of the target vehicle. It was concluded that the fusion was accurate. Furthermore, in the only vision case, the lateral and longitudinal errors were similar to the vision + LiDAR case. The lateral error increased from Scenarios (a) to (c), but it was 1.4343 m. It was smaller than the half-width of the lane, so there was no difficulty in distinguishing the lane where the target vehicle was maneuvering.

### 3.2. CIPV

#### 3.2.1. Scenario

**Figure 11:**
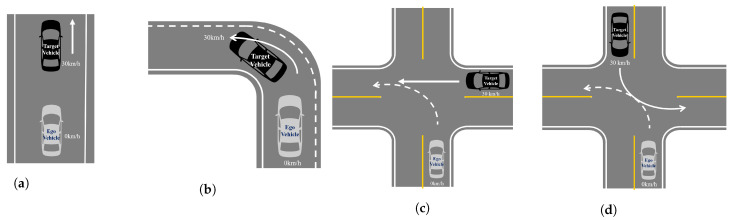
CIPV scenario. The dashed arrow presents the path of the ego vehicle, and the solid arrow presents the path of the target vehicle. (**a**) Straight road. (**b**) Left turn road. (**c**) Turn across the path. (**d**) Turn across the path.

In this scenario, the experiment was conducted by assuming that there was information about the path. Actually, in the case of the curved path in the experiment, the path data consisting of the GPS-based UTM coordinate system were used. However, as for the case of the straight path, the center point, which was the result of the deep learning-based lane detection algorithm ENet-SAD, was used [23]. Since the data of the path were the values of the BEV coordinate system, the process of determining whether the recognized vehicle belonged to the path was also performed in the BEV coordinate system. Thus, the BEV coordinate system of the detected vehicle was considered as an essential one. When the target vehicle was recognized through vision, we used the transformed result value. Therefore, the transform process through vision showed that this process was also considered as another essential part of this study. As shown in Figure 11, four scenarios were prepared to confirm the detection performance on a straight road and a curved road, which are part of the ACC performance evaluation scenarios presented by ISO [12].

#### 3.2.2. The CIPV Result of the Scenario

**Figure 12:**
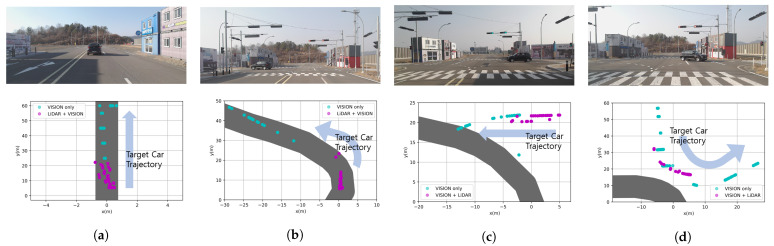
Result of the CIPV scenario. The part colored in solid gray shows the path of the ego vehicle. In scenarios (**a**–**d**), LiDAR only detects when it is near, but in the case of vision, it can be seen that it is recognized well even when it is far away.

While proceeding to the four scenarios in Figure 11, the result of recognizing the target vehicle and path data was created in the BEV coordinate system. The BEV coordinate system means the detection coordinates when viewed in the xy plane from a place perpendicular to the z-axis. In the only vision case, the BEV data form of the fusion data was [x1,y1,x2,y2], and the cyan dots in Figure 12 are $\frac{x1+x2}{2}$, $\frac{y1+y2}{2}$. In the LiDAR+vision case, the BEV data form was [x1,y1,x2,y2,x3,y3,x4,y4], and the cyan dots in Figure 12 are $\frac{x1+x2}{2}$, $\frac{y1+y2}{2}$, where y1,y2 is smaller than y3,y4. In the coordinate system, (0, 0) is the location of the ego vehicle that is in a stopped state, as shown in Figure 11. Moreover, the shaded part in gray color shown in Figure 12 is the result of connecting the paths. Firstly, in the case of a straight line, it is the center point obtained through ENet-SAD. Secondly, in the case of a curve, the point value is drawn through the GPS UTM coordinates. In Figure 11a scenario, the ego vehicle was stopped and the target vehicle was moving away in a straight lane. At this time, the result of recognizing the target vehicle is shown in Figure 12a, and it can be confirmed that the LiDAR recognized only from near and did not recognize when it was further away. In the scenario (b) of Figure 11, the ego vehicle was stopped and the target vehicle proceeded to the left curve. Originally, when the road to which the ego vehicle was going was the left curve, we want to check whether the recognition result comes into the path, and it can be seen that it recognizes the CIPV well as shown in Figure 12b. As shown in Figure 12c, if there is no BEV result transformed through vision, as the target vehicle proceeds from right to left, we note that it is not possible to recognize the situation where the ego vehicle is overlapping with the path in which it proceeds. In the scenario (d) of Figure 11, the path of the ego vehicle was the left curve, whereas the path of the target vehicle was completely different. It is very important to recognize the target vehicle as a vehicle outside of the path so that it does not overlap with the path to go. As a result, as shown in Figure 12d, it can be seen that the result of fusion is accurate enough to represent the trajectory of the target vehicle, and it is confirmed that it does not overlap with the path of the ego vehicle.

As shown in Figure 12, we show that the limitation of the low-channel LiDAR was able to be overcome with the result of sensor fusion. Through sensor fusion, the data recognized as a car could be continuously recognized from a distance, and the BEV value was obtained in any situation recognized by LiDAR or vision, whether it was far away or near. In addition, as a result, it was possible to select the nearest target vehicle in the path by fusion with the path in the BEV coordinate system, and then, it was used to check the performance by the ACC test.

### 3.3. ACC

#### 3.3.1. Scenario

**Figure 13:**
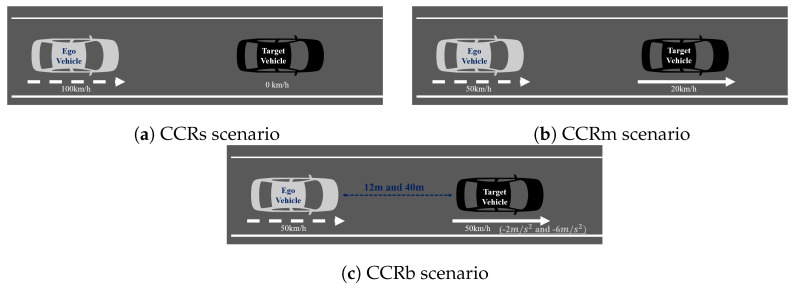
Euro NCAP AEB test protocol scenario. (**a**) CCRs scenario. (**b**) CCRm scenario. (**c**) CCRb scenario.

If the speed of the CIPV and the speed of the ego vehicle are kept similar, only LiDAR is sufficient because the change in speed is insignificant even when the CIPV is at a close distance. Therefore, a situation that requires the recognition of a distant object and a quick response was selected to evaluate the performance of sensor fusion. By referring to Euro NCAP’s AEB test protocol scenario, we checked whether the vehicle decelerated properly in three cases: CCRs, CCRm, and CCRb. Figure 13a is a test to check the function of stopping after recognizing the target vehicle while proceeding at 100 km/h toward the vehicle stopped in front. Figure 13b is a test to check the function of decelerating after recognizing the target vehicle while proceeding at 50 km/h toward a vehicle running at a low speed ahead. Lastly, Figure 13c is a test to validate the braking function when the target vehicle in front suddenly stops after recognizing the vehicle while proceeding at 50 km/h toward the vehicle running at the same speed ahead.

#### 3.3.2. The Result of the Scenario

- CCRs

**Figure 14:**
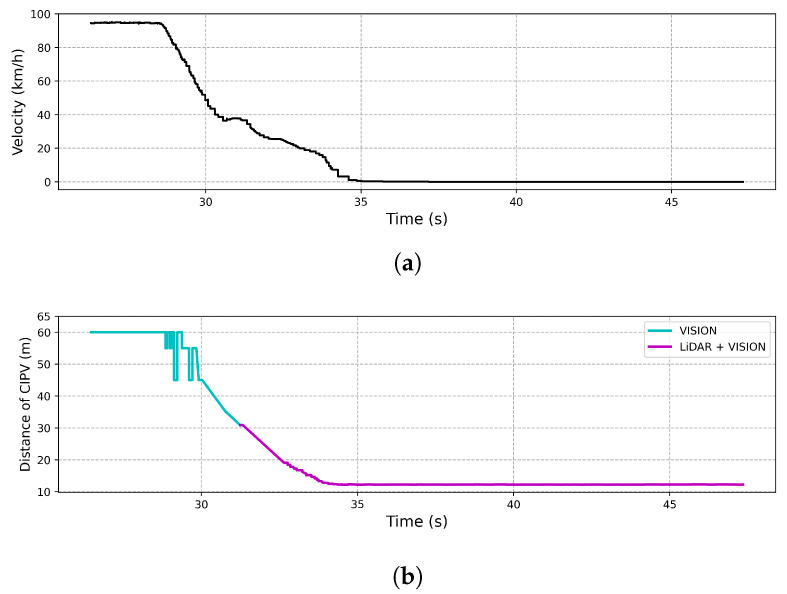
Result of CCRs. (**a**) The velocity of the vehicle by time. (**b**) The distance to the CIPV by time.

Figure 14 shows the result of an experiment on the response to an ego vehicle in front of a stationary target vehicle during ACC driving at a speed of 100 km/h. As shown in Figure 14, when an ego vehicle approached 30 m from the target vehicle, LiDAR and vision were able to recognize the target vehicle simultaneously, while only the vision sensor recognized the target vehicle beyond 30 m. In order to start deceleration from 30 m using only LiDAR and stop at a distance of 10 m, an average deceleration rate of at least 19.29 m/s${}^{2}$ was required. However, we note that the ego vehicle stopped at a distance of 10 m with an average deceleration rate of 7.72 m/s${}^{2}$ because the target vehicle was recognized as the CIPV through the result of sensor fusion.

- CCRm

**Figure 15:**
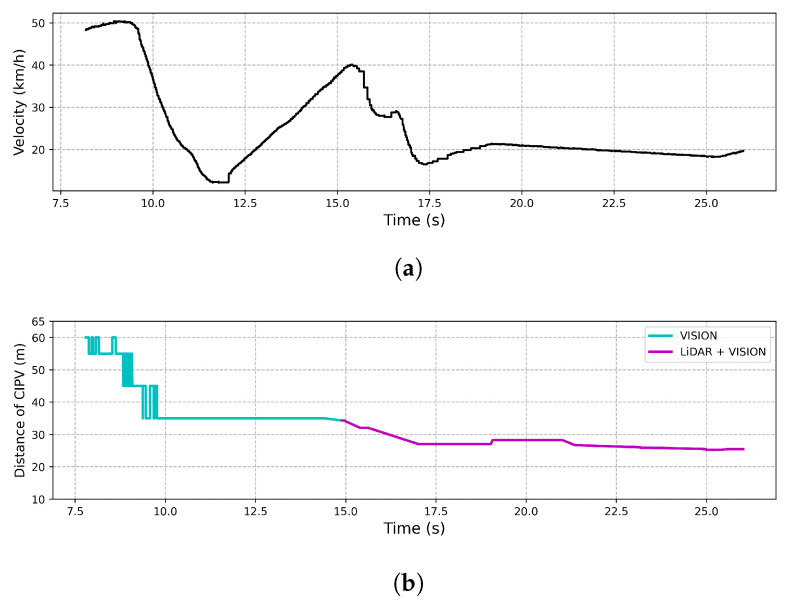
Result of CCRm. This test aimed to keep the distance from the preceding vehicle at 20 m, and the speed of the preceding vehicle was kept constant at 20 km/h. (**a**) The velocity of the vehicle by time. (**b**) The distance to the CIPV by time.

As shown in Figure 15, the result of the experiment also described the ego vehicle response to the target vehicle traveling at a low speed during ACC driving at a speed of 50 km/h. In Figure 15, when the ego vehicle approached about 35 m from the target vehicle, it was detected by LiDAR and vision at the same time, and before that, the target vehicle was detected by only vision beyond 35 m. If the ego vehicle started deceleration from 35 m using only LiDAR, it needed an average deceleration rate of 5.401 m/s${}^{2}$ to drive at the vehicle speed ahead. On the other hand, as shown in Figure 15, it reached 20 km/h with an average deceleration rate of 2.025 m/s${}^{2}$. Overshoot occurred in the speed graph, but since it was an experiment to see the applicability of the proposed method by initial recognition distance and response speed, the overshoot was not considered. This result showed that the CIPV was discovered from a distance while driving through the proposed sensor fusion, which made the ego vehicle respond quickly.

- CCRb

**Figure 16:**
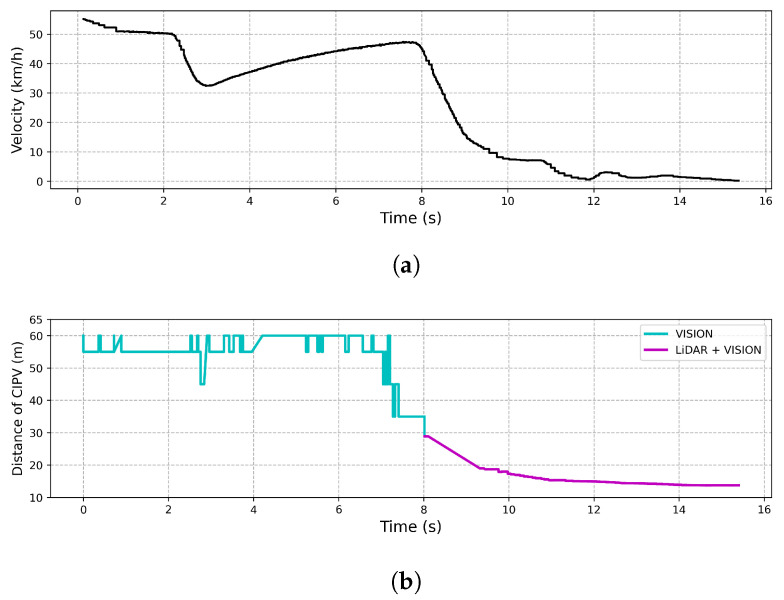
Result of CCRb. (**a**) The velocity of the vehicle by time. (**b**) The graph shows the distance to the CIPV by time.

Figure 16 shows the result of an experiment on the response to a target vehicle in front that suddenly stops while driving at 50 km/h. In Figure 16, when the target vehicle was approached by 30 m, the target vehicle was detected by LiDAR and vision simultaneously, while only the vision sensor was used to recognize the preceding vehicle beyond 30 m. In order to start deceleration from 30 m using only LiDAR and stop at a distance of 10 m, an average deceleration rate of 4.823 m/s${}^{2}$ or more than the average was required. However, as shown in Figure 16, it stopped at a distance of 10 m with an average deceleration rate of 2.411 m/s${}^{2}$. Overshoot occurred in the speed graph, but since it was an experiment to see the utility of the initial recognition distance and response speed, the overshoot was not considered. Thus, we note that the proposed algorithm achieved a much smoother response because the CIPV was being tracked more than 50 m away while driving through fusion result.

## 4. Conclusions

In this paper, we proposed a sensor fusion method that utilized LiDAR and vision-tracked data and improved the detection of the CIPV by transforming the pixel image coordinates to the BEV. The location of the target vehicle was not known through the tracking results of vision. Therefore, in this paper, a projection equation that fit the point clouds of LiDAR to the image was experimentally determined, and the pixel image coordinates were transformed to the BEV by using that equation inversely. As a result of tests using the target vehicle in a straight lane and a side lane for the ego vehicle, it showed an error of 1.5895 m laterally and 0.7408 m longitudinally at a distance of about 45 m in front, which was able to distinguish the lane where the target vehicle was located. Afterwards, all these data were fused, and the results were used for the performance evaluation of the detection the CIPV, as well as the ACC performance evaluation. Thus, we verified that the proposed sensor fusion algorithm was able to improve the performance of the recognition of the preceding target vehicle in various situations that commonly occur in straight lanes, curved lanes, and intersections. Consequently, as a test result of applying the fusion data to ACC, we concluded that the proposed algorithm accomplished a convincing test result of the performance of the braking system, which made the ego vehicle twice as smooth as the case of using only LiDAR. In future work, more ACC tests equipped with a fine-tuned lower level controller will be performed by the proposed sensor fusion algorithm in diverse scenarios.

## Figures and Tables

**Table 1:** Sensors’ specification.

Sensor	Product Name	Specification
LiDAR	Velodyne Puck LiDAR (previously VLP-16)	16 channels, measurement range up to 100 m with 10 fps
Camera	Logitech StreamCam	FoV 78, resolution 720p with 60 fps
GPS	RTK GNSS GPS (MRP-2000)	resolution 0.010m with 10 fps

**Table 2:** Fusion data. In only the vision case, “bird’s eye-view”, “object’s closest point”, “distance” and “in-path” data are provided as the fusion data only when the ID type corresponds to “car”.

Data Type	Only Vision	LiDAR + Vision	Only LiDAR
Name	“V”	“VL”	“L”
Bounding box 2d	[x1, y1, x2, y2]	Choose the LiDAR data	[x1, y1, x2, y2]
Bird’s eye-view	[x1, y1, x2, y2]	Choose the LiDAR data	[x1, y1, x2, y2, x3, y3, x4, y4]
Object’s closest point	[x, y, z]	Choose the LiDAR data	[x, y, z]
Distance	*meters*	Choose the LiDAR data	*meters*
Velocity	-	Choose the LiDAR data	*meter per s* ${}^{2}$
In-path	0 or 1	Choose the LiDAR data	0 or 1
Moving state	-	Choose the LiDAR data	0 or 1
Type ID	Result of YOLOv3	Choose the vision data	-
Time to collision	-	Choose the LiDAR data	*s*

**Table 3:** Error of the BEV from the fusion data.

Type of Path	Type of Fusion Data	Distance Error (m)↓			Lateral Error (m)↓			Longitudinal Error (m)↓		
		Min	Max	MAE	Min	Max	MAE	Min	Max	MAE
Scenario (a)	vision + LiDAR	0.0010	0.4999	0.2767	0.0286	1.3587	0.3764	0.0025	1.3587	0.1625
	only vision	0.0077	0.4990	0.2464	0.0704	2.9954	0.4567	0.0024	2.9954	0.1902
Scenario (b)	vision + LiDAR	0.0031	0.0957	0.0498	0.6957	1.1594	0.9437	0.0001	1.1594	0.0697
	only vision	0.0045	0.4957	0.2424	0.1050	3.4802	0.8706	0.0011	3.4802	0.2029
Scenario (c)	vision + LiDAR	0.0033	0.0967	0.0532	0.1697	6.4679	3.0378	0.0309	6.4649	0.1857
	only vision	0.0041	0.4821	0.2432	0.0572	5.4662	1.4343	0.0034	5.4662	0.2386
